# Severe Hypernatremia as Presentation of Netherton Syndrome

**DOI:** 10.1055/s-0043-1776983

**Published:** 2023-11-22

**Authors:** A. Di Nora, M.C. Consentino, G. Messina, T. Timpanaro, P. Smilari, P. Pavone

**Affiliations:** 1 Department of Clinical and Experimental Medicine, University of Catania, Catania CT, Italy

**Keywords:** hypernatremia, genodermatosis, *Trichorrhexis invaginata*

## Abstract

Netherton syndrome is a rare, multisystem, autosomal recessive genodermatosis characterized by a triad of manifestations: congenital ichthyosis, immune dysregulation, and scalp anomalies. We report the case of a 1-month-old male infant evaluated for failure to thrive and feeding difficulties. At birth, the infant was admitted to intensive care for severe hypernatremia (natremia 186 mg/dL). Upon entering the ward, the general conditions were poor. He presented with diffuse erythrodermia. A dermatological evaluation showed evidence of “invaginated trichuriasis,” a typical sign of Netherton syndrome. Netherton syndrome is caused by a genetic mutation causing loss of function of the SPINK5 gene it encodes for the LEKTI protein, normally expressed in epithelia. Loss of LEKTI induces severe skin barrier defect. The history of the disease is characterized by serious potential complications in the first months of life, such as the risk of hypernatremic dehydration induced by high skin permeability, recurrent and/or severe infections, and growth retardation.

## Introduction

Netherton syndrome (NS) is a rare, multisystemic, autosomal recessive genodermatosis characterized by a triad of manifestations: congenital ichthyosiform erythroderma, hair shaft abnormalities, and immune dysregulation. The incidence of the disorder is evaluated to be 1/200,000, and the prevalence is 1 to 9/1,000,000. 1 2 The genetic defect involves a serine protease inhibitor Kazal-type 5 (SPINK5) gene, encoding lymphoepithelial Kazal-type–related inhibitor (LEKTI), expressed in hair follicles and the granular layer of the epidermis. 3 4 Deficiencies or anomalies in LEKTI are responsible for an excessive serine protease activity, causing the premature stratum corneum detachment and defect of skin barrier function. 3 4

Comel in 1949 described one of the main characteristics of NS, namely, ichthyosis linearis circumflexa, that consists of pruritic polycyclic erythematous patches with a double-edged circinate or serpiginous scale. 5 Later, Netherton in 1958 described the pathognomonic hair abnormality of NS, the trichorrhexis invaginata (bamboo hair). 6 Since then, several years have passed and NS is still a diagnostic challenge for the clinicians. In addition, there are no specific therapies yet. 7 An early diagnosis is crucial to start the correct management of these patients, due to a significant mortality in the first years of life. 7 Thus, neonatologists and pediatricians should consider this rare but potentially fatal diagnosis in the clinical practice. Here, we report the case of a 1-month-old male infant, admitted to our center for failure to thrive and feeding difficulties. The perinatal history was characterized by hypernatremia and ichthyosiform erythroderma. The clinical features, the genetic analyses, and the actual management were also reported.

## Case Report

A 1-month-old male infant was evaluated for failure to thrive and feeding difficulties. He was delivered at 36 weeks after pregnancy with SARS-CoV-2 infection at the third trimester and gestational diabetes treated with alimental diet. Familial and antenatal history was unremarkable. Parents declared to be not familiars. At birth, the infant did not have asphyxiation or jaundice. Birth weight was 2,580 g. After 3 days, he was admitted in the neonatal intensive care unit for dehydration and skin changes. Electrolytes analyses showed hypernatremia (186 mmol/L), treated with water replacement. A dermatological consilience revealed a desquamative erythroderma and subsequent seborrheic dermatitis. He was discharged at 2 weeks of life, with normal value of natremia and indication of breastfeeding. Two weeks later, he was admitted to our pediatric center “Policlinico G. Rodolico” in Catania, Italy. At the time of admission, the infant presented with diffuse erythroderma, skin coat with large areas of flaking that in the region of the scalp took on a crusty appearance with areas of alopecia. The subcutaneous tissue was poorly represented; muscle masses appeared hypotrophic and hypotonic. (rhythmic heart sounds, systolic murmur 2/6; flat abdomen of regular shape and volumes, treatable and painless; sensory awake valid crying, normal elicitable osteotendineous rotouleous reflex with axial and appendicular hypotonia; weight 2,670 g). Routine analyses revealed positive value of polymerase chain reaction, such as in systemic infections. Skin swabs were performed, revealing the presence of *Pseudomonas aeruginosa* . Treatment with Merrem and Teicoplanin was started, with a good response. A new dermatological consultation was performed, describing “abundant lamellar desquamation with the presence of skin aspects suggestive of circumflex linear ichthyosis.” The video-dermatoscopic examination of the scalp revealed the presence of invaginated trichuriasis. Genetic analyses were performed, confirming the suspect of NS. It revealed the mutation c.238dup (p.Ala80fs) in homozygosis of SPINK5, localized on chromosome 5q32. Genetic analysis in the parents revealed the same mutation in heterozygosis in both.

## Discussion

**Fig. 1:**
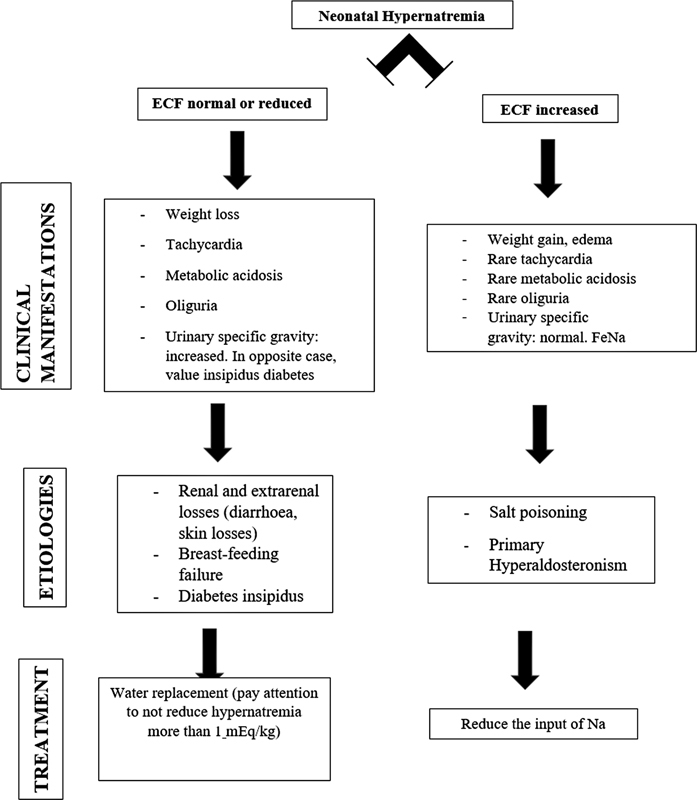
Proposal of a flowchart in cases of neonatal hypernatremia. ECF, extracellular fluid; FeNa, fractional excretion of sodium.

Netherton syndrome is caused by loss-of-function mutations in the SPINK5 gene coding for LEKTI, usually expressed in stratified epithelia. 8 Loss of LEKTI induces a severe skin barrier defect. The history of the disease is characterized by potential severe complications in the first months of life, such as the risk of hypernatremic dehydration induced by high skin permeability, recurrent and/or severe infections, and failure to thrive. 9 Jones et al in 1986 reported the occurrence of hypernatremia in two neonates with NS. 10 Recently, in 2020 Bellon et al reported the clinical experience of a series of 43 children affected by NS. They found hypernatremia in 53.5% of the population, mostly children characterized by skin inflammation at birth. 9 Neonatal hypernatremia is an uncommon finding and it has been frequently associated with breastfeeding failure and weight loss. 10 11 12 In our case, the extreme hypernatremia could not fully be explained only by weight loss. Other differential diagnoses were salt poisoning (rare in a young infant), diarrhea, and polyuria (in cases of diabetes insipidus; Fig. 1 ) We believe that the combination of inadequate breast milk and excess water losses due to the congenital erythroderma was the direct cause in our case.

**Fig. 2:**
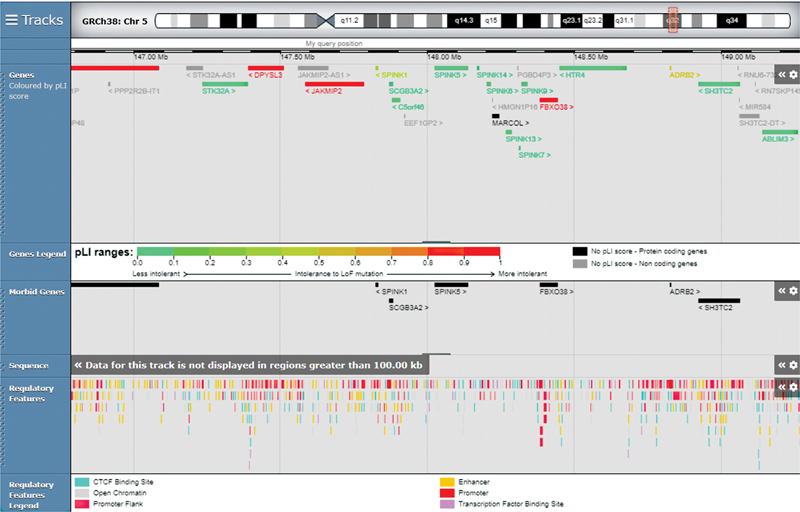
Modified from *SFARI* genes, you can see SPINK5 involved with high confidence.

Clinically, the evaluation under the microscope or with trichoscopy revealed the pathognomonic sign of trichorrhexis invaginate, giving the doubt of NS. In addition, failure to thrive was a confirmation of systemic substance pathology. Genetic analysis confirmed the clinical suspect, revealing the mutation c.238dup (p.Ala80fs) in homozygosis of SPINK5, localized on chromosome 5q32 ( Fig. 2 ). As reported in ClinVar, this sequence change creates a premature translational stop signal (p.Ala80Glyfs*19) in the SPINK5 gene, causing an absent or disrupted protein product. This variant is present in population databases (rs766893577, ExAC 0.002%) and it has been observed in several individuals affected with NS (PMID: 22089833, 25917539, 16628198, 10835624). 13 14 15 16 17 In our case, the genetic analysis in both parents revealed the variant in heterozygosis. As reported in literature, genetic analysis in NS is mandatory not only for the diagnosis but also for the management and the prognosis. 9 18 In fact, the various SPINK5 mutations are associated with organ-specific expression profiles for the various isoforms of LEKTI. Sarri et al conducted an interesting study analyzing the relationship between genotype and phenotype in patients with NS. 18 They concluded that mutations located more upstream in LEKTI will produce a more severe phenotype than similar mutations located toward the 3′ region. 18 About the genetic mutation of our proband, in literature it has been correlated to the increased susceptibility to skin carcinomas, with a high association between HPV and non-melanoma skin cancers. 16

Infections are often reported in patients with NS. Bellon et al found cutaneous bacterial infections (mainly *Staphylococcus aureus* and *Pseudomonas aeruginosa* ) in 27 among 43 children affected by the disease. 9 It emphasizes that NS is a systemic genodermatosis that requires rigorous care procedures especially in the first year of life. In addition, comorbidities such as gastrointestinal, endocrinal, pulmonary involvement are reported in literature, with high incidence of severe metabolic anomalies. 9 The failure to thrive and chronic non-infectious diarrhea, observed in severe NS forms, might be partly related to abnormal digestive permeability and absorption as SPINK5 mRNAs are expressed in the gastrointestinal tract. 19 20

About the management, in literature hydration up to >300 mL/kg/day is recommended, instead up to ∼160 mL/kg/day in healthy newborn. 9 Increased calorie and protein needs for adequate growth were required in the most severe patients (220 kcal/kg/day and 5 g/kg/day, respectively, compared to 90–110 kcal/kg/d and 1–2 g/kg/d in the healthy newborn). 9 In a recent guideline, recommended treatment is topical therapy. Emollients are indicated at least two times a day and after bathing. 21 Multiple advances in novel therapies for NS are currently in progress. Because of the known increased activity of KLK 5 in NS, several specific inhibitors of KLK 5 are under development. 22

## Conclusions

NS is a rare genodermatosis. Its early diagnosis is important to start the best management. Hypernatremia and systemic infections are possibly fatal complications. Trichoscopy is an accessible and noninvasive auxiliary diagnostic tool in cases of doubt. Genetic test is mandatory for the diagnosis and useful to define the prognosis of the patient affected.
